# Application of MinION Amplicon Sequencing to Buccal Swab Samples for Improving Resolution and Throughput of Rumen Microbiota Analysis

**DOI:** 10.3389/fmicb.2022.783058

**Published:** 2022-03-24

**Authors:** Hiroto Miura, Masayuki Takeda, Megumi Yamaguchi, Yoshihisa Ohtani, Go Endo, Yasuhisa Masuda, Kaede Ito, Yoshio Nagura, Kunihiro Iwashita, Tomohiro Mitani, Yutaka Suzuki, Yasuo Kobayashi, Satoshi Koike

**Affiliations:** ^1^Graduate School of Agriculture, Hokkaido University, Hokkaido, Japan; ^2^National Livestock Breeding Center, Fukushima, Japan; ^3^Mito Research Center, Meiji Feed. Co., Ltd, Ibaraki, Japan; ^4^Field Science Center for Northern Biosphere, Hokkaido University, Hokkaido, Japan

**Keywords:** rumen microbiota, buccal swab, MinION, amplicon sequencing, 16S rRNA gene

## Abstract

The Illumina MiSeq platform has been widely used as a standard method for studying the rumen microbiota. However, the low resolution of taxonomic identification is the only disadvantage of MiSeq amplicon sequencing, as it targets a part of the 16S rRNA gene. In the present study, we performed three experiments to establish a high-resolution and high-throughput rumen microbial profiling approach using a combination of MinION platform and buccal swab sample, which is a proxy for rumen contents. In experiment 1, rumen contents and buccal swab samples were collected simultaneously from cannulated cattle (*n* = 6) and used for microbiota analysis using three different analytical workflows: amplicon sequencing of the V3–V4 region of the 16S rRNA gene using MiSeq and amplicon sequencing of near full-length 16S rRNA gene using MinION or PacBio Sequel II. All reads derived from the MinION and PacBio platforms were classified at the species-level. In experiment 2, rumen fluid samples were collected from beef cattle (*n* = 28) and used for 16S rRNA gene amplicon sequencing using the MinION platform to evaluate this sequencing platform for rumen microbiota analysis. We confirmed that the MinION platform allowed species-level taxa assignment for the predominant bacterial groups, which were previously identified at the family- and genus-level using the MiSeq platform. In experiment 3, buccal swab samples were collected from beef cattle (*n* = 30) and used for 16S rRNA gene amplicon sequencing using the MinION platform to validate the applicability of a combination of the MinION platform and buccal swab samples for rumen microbiota analysis. The distribution of predominant bacterial taxa in the buccal swab samples was similar to that in the rumen samples observed in experiment 2. Based on these results, we concluded that the combination of the MinION platform and buccal swab samples may be potentially applied for rumen microbial analysis in large-scale studies.

## Introduction

As the world population is increasing rapidly, food security has become a matter of major global concern. Improvement of livestock production efficiency with low adverse environmental impact is required to ensure stable supply of meat and milk, which are important sources of animal protein (Huws et al., 2018). Ruminant animals mainly depend on rumen microbes to acquire nutrients from the feed, particularly from forage. Additionally, recent studies have suggested that individual differences in rumen microbial composition influence host productivity, such as feed efficiency (Li et al., 2019), milk composition (Tong et al., 2018; Xue et al., 2020), and methane yield (Kittelmann et al., 2014; Ramayo-Caldas et al., 2020). These findings indicate that ruminant productivity can be enhanced by controlling the distribution of specific rumen microbes. Therefore, the rumen microbiota is a potential target that can be manipulated to improve animal productivity (Huws et al., 2018). However, the details regarding the distribution of key microbes linked to host productivity and their mode of action remain unclear. The key microbes that influence host productivity have to be determined using large-scale data sets for improving animal productivity *via* modulation of the microbiota. In addition, the resolution of taxonomic identification in microbiota analysis has to be increased from the genus to species-level to clarify the function of the key players. Therefore, a method for high-throughput and high-resolution rumen microbial profiling is required.

The method for collecting samples for a large-scale data set is the major bottleneck in rumen microbiota studies. Common sampling methods, such as oral intubation of the stomach tube and fitting of the rumen cannula, are considered impractical for large-scale studies because of their laborious and invasive nature (Duffield et al., 2004; Shen et al., 2012). To overcome this limitation, recent studies have shown that the buccal swab samples of ruminants can be used as a proxy for rumen contents, which is practically feasible for large-scale studies (Kittelmann et al., 2015; Tapio et al., 2016; Young et al., 2020; Amin et al., 2021).

Analysis of parts of the 16S rRNA gene using the Illumina MiSeq platform has been widely used as a standard method in studies on the rumen microbiota (McGovern et al., 2018). Although MiSeq amplicon sequencing has revealed the structure of the rumen microbiota, its use is limited by low resolution owing to the targeting of parts of the 16S rRNA gene (Myer et al., 2016). In the last few years, new platforms that allow the sequencing of full-length 16S rRNA gene amplicons, such as MinION and PacBio Sequel II system (PacBio), have been introduced to improve the resolution of taxonomic identification in microbiota studies. Notably, the MinION platform has advantages, such as affordable introduction costs, simplicity, and sequencing speed (Ciuffreda et al., 2021). Although currently the error rate of MinION (5%) is higher than that of MiSeq (0.1%), the sequencing reads of full-length 16S rRNA genes containing multiple variable regions derived from the MinION platform complement high error rate and enable taxonomic identification of bacterial composition at the species-level (Benítez-Páez et al., 2016). Recently studies have revealed that the MinION platform provides greater taxonomic resolution than short-read sequencing in the human gut (Matsuo et al., 2021), mouse gut (Shin et al., 2016), and building dust (Nygaard et al., 2020). However, it has not yet been validated for the analysis of rumen microbiota.

In the present study, we conducted a trial to establish a high-resolution and high-throughput rumen microbial profiling approach using a combination of the MinION platform and buccal swab sample, which is a proxy for rumen contents (experiment 1). In addition, we applied the newly established approach to rumen samples (experiment 2) and buccal swab samples (experiment 3) to validate the applicability of this combination for characterizing the rumen microbiota.

## Materials and Methods

We conducted three animal experiments in this study. Buccal swabs and rumen contents were collected from cattle and used for amplicon sequencing using MiSeq, MinION, and PacBio platforms. The animal protocols were in accordance with the Guidelines for Animal Experiments and the Act on Welfare and Management of Animals, Hokkaido University, and the Principles and Guidelines for Animal Use set by the National Livestock Breeding Center. The animal experimental procedures used in the present study were approved by the Animal Care Committee National Livestock Breeding Center (Authorized Number: 31-29).

## Experiment 1: Trial to Establish High-Resolution and High-Throughput Rumen Microbial Profiling Approach Using Combination of MinION Platform and Buccal Swab Sample

### Sample Collection and DNA Extraction

Three dry Holstein dairy cows (142.7 ± 49.6 months old) reared in the experimental farm of the Hokkaido University (Hokkaido, Japan) and three fattening Japanese Black cattle (37.1 ± 0.5 months old) reared in the experimental farm of the Mito Research Center, Meiji Feed Co., Ltd. (Ibaraki, Japan) were used in experiment 1. The animals were fitted with a ruminal cannula. Holstein cows were fed Timothy hay (3–5 kg/day) and corn silage (20 kg/day), and Japanese Black cattle were fed rice straw (2 kg/day) and concentrate diet (6 kg/day). Animals were fed once daily at 10.00 h. The animals had free access to mineral blocks and water. The aim of experiment 1 was not to compare the microbiota between the animal groups but to confirm whether the combination of buccal swabs and MinION platform can be applicable to the animals fed different diets, which leads to changes in microbiota composition.

Buccal swab samples were collected from the mouth of the animals 3 h after feeding. Three cotton swabs were inserted into the oral cavity of each animal and gently swabbed across the inner side of the cheek for approximately 5 s. The buccal swabs were then placed in a sterile 15 ml polypropylene tube containing 2 ml nucleic acid preservation buffer (Camacho-Sanchez et al., 2013) and stored on ice during sampling. Immediately after buccal swab sampling, the rumen contents were collected from individual animals *via* the rumen cannula. The collected rumen contents were then stored on ice during sampling without separating the solid and liquid fractions. After shipping to the laboratory, all samples were stored at −80°C until use.

Microbial DNA was extracted and purified from buccal swabs (three cotton swabs per animal) and rumen contents (250 μl/animal) using the repeated bead beating plus column method (Yu and Morrison, 2004). Buccal swabs or rumen contents were homogenized with 0.4 g of sterile glass beads (0.3 g of 0.1 mm and 0.1 g of 0.5 mm) and 1 ml cell lysis buffer (500 mM NaCl, 50 mM Tris-HCl, pH 8.0, 50 mM EDTA, and 4% sodium dodecyl sulfate). Lysates were then incubated at 70°C for 15 min, and the supernatant was collected for further process. Bead beating and incubation steps were repeated once, and all supernatants were combined. Total DNA was precipitated with 10 M ammonium acetate and isopropanol followed by purification using a QIAamp Fast DNA Stool Mini Kit (Qiagen, Hilden, Germany). DNA concentration was quantified using a NanoDrop 2000 spectrophotometer (Thermo Fisher Scientific, Waltham, MA, United States). Microbial DNA from rumen samples was diluted to a final concentration of 5 ng/μl for further analysis. Owing to the low concentration of DNA from the buccal swab (<1 ng/μl), DNA solution was used for analysis without dilution.

### Amplicon Sequencing and Data Analysis

#### MiSeq Platform

Microbial DNA was used to amplify the V3–V4 region of the 16S rRNA gene using the primer set S-D-Bact-0341-b-S-17 (5′-CCT ACG GGN GGC WGC AG-3′) and S-D-Bact-0785-a-A-21 (5′-GAC TAC HVG GGT ATC TAA TCC-3′; Herlemann et al., 2011). The PCR mixture consisted of 12.5 μl of 2× KAPA HiFi HotStart Ready Mix (Roche Sequencing, Basel, Switzerland), 5.0 μl of each primer (1.0 μM), and 2.5 μl DNA. PCR was performed as follows: initial denaturation at 95°C for 3 min, followed by 25 cycles of 95°C for 30 s, 55°C for 30 s, and 72°C for 30 s, and a final extension step at 72°C for 5 min. Amplicons were purified using AMPure XP beads (Beckman Coulter, Brea, CA, United States), followed by 2 × 300 bp paired-end sequencing on the MiSeq platform (Illumina, San Diego, CA, United States) using MiSeq reagent kit v3.

Data obtained from the MiSeq platform were analyzed using the QIIME2 version 2020.8 (Bolyen et al., 2019). Paired-end reads were merged using q2-dada2 plugin (Callahan et al., 2016) to generate amplicon sequence variants (ASVs). The taxonomic classification of ASVs was performed at the species-level using the Naive Bayes classifier with the q2-classifier sklearn plugin against the SILVA 138 99% reference data set.

#### MinION Platform

Microbial DNA was used to amplify the near full-length (V1–V9 region) of 16S rRNA gene using four-primer PCR with rapid adapter attachment chemistry, following the instructions provided by Oxford Nanopore Technologies (Oxford, United Kingdom). The primer sets 27F (5’-“AGA GTT TGA TCC TGG CTC AGA”-3’) and 1525R (5’-“AAG GAG GTG WTC CAR CC”-3’; Lane, 1991) were used to amplify the V1–V9 region of the 16S rRNA gene. The PCR mixture consisted of 25.0 μl of 2× KAPA HiFi HotStart Ready Mix (Roche Sequencing), 50 nM of each inner primers, and 1.5 μl barcoded outer primers from the PCR barcoding kit (SQK-PBK004; Oxford Nanopore Technologies), and 6.0 μl DNA. PCR was performed according to the following program: initial denaturation at 94°C for 1 min, 5 cycles at 94°C for 30 s, 63°C for 30 s, and 65°C for 90 s; 30 cycles at 94°C for 30 s, 62°C for 30 s, and 65°C for 90 s; final extension step at 65°C for 5 min. Amplicons from each sample were purified using AMPure XP beads (Beckman Coulter). To remove short fragments, the ratio of samples to beads (50: 30) was optimized according to the recommendation of Beckman Coulter. For DNA library preparation, the purified amplicons were mixed at equal concentrations and diluted to 5 fmol/μl, followed by incubation with 10 μl of mixed amplicon and 1 μl of Rapid Adapter (SQK-PBK004; Oxford Nanopore Technologies) at room temperature for 5 min. The prepared DNA library (11 μl) was mixed with 25.5 μl loading beads and 34 μl sequencing buffer (SQK-PBK004; Oxford Nanopore Technologies), and the volume was made up to 75 μl with sterilized dH_2_O. The entire DNA library (75 μl) was loaded onto the R9.4 flow cell (FLO-MIN106; Oxford Nanopore Technologies) for sequencing. The MINKNOW software v.3.4.8 (Oxford Nanopore Technologies) was used for data acquisition.

The Fast5 formatted sequences from MINKNOW were basecalled to Fastq-formatted sequences and then demultiplexed with the Guppy software v.4.4.2 (Oxford Nanopore Technologies). The adapters and barcodes were trimmed using Porechop v.0.2.3 (Oxford Nanopore Technologies). The sequences were filtered by length and mean quality score using NanoFilt (v.2.7.1) based on the following criteria: sequences with average quality score of 9 or above and length of 1,300–1,600 bp were retained. The filtered sequences were aligned against the SILVA 138 99% reference data set using the LAST aligner (v.874) using the following parameters: -r 1 -q 1 -a 1 -b 1 (match score of 1, mismatch cost of 1, gap opening cost of 1, and gap extension cost of 1) according to previous studies (Quick et al., 2014; Shin et al., 2016; Nygaard et al., 2020). The highest scoring alignment was retained for each read. Taxonomic IDs with only one aligned sequence read (i.e., singletons) were excluded from the analysis.

#### PacBio Platform

Microbial DNA was used for the amplification of the near full-length (V1–V9 region) 16S rRNA gene using the primer set 27F (5′-AGR GTT YGA TYM TGG CTC AG-3′) and 1492R (5′-RGY TAC CTT GTT ACG ACT T-3′; Weisburg et al., 1991) with sample-specific barcodes per the instructions of PacBio (Menlo Park, CA, United States). The PCR mixture consisted of 12.5 μl of 2× KAPA HiFi HotStart Ready Mix (Roche Sequencing), 1.5 μl dH_2_O, 3.0 μl of each primer (2.5 μM), and 5 μl DNA. The PCR steps were performed according to the following program: initial denaturation at 95°C for 3 min, followed by 20 cycles of 95°C for 30 s, 57°C for 30 s, and 72°C for 60 s, and a final extension step at 72°C for 5 min. Amplicons were purified using AMPure PB beads, followed by sequencing on the Sequel II system with SMRTbell® Express Template preparation kit 2.0 (PacBio).

Raw reads obtained from the PacBio platform were demultiplexed using lima application (v.1.10.0). The circular consensus reads (ccs) were then generated using the SMRT Link software (v.5.13) based on the following criteria: minimum predicted accuracy = 0.995, minimum number of passes = 3, minimum length of reads, 1,300 bp; and maximum length of reads, 1,600 bp. The filtered sequences were then used for taxonomic classification using the LAST aligner as the same manner as MinION sequencing data analysis.

#### Validation of Analyzed Region of 16S rRNA Gene and Analytical Software

Previous studies have demonstrated that the results of amplicon sequencing in microbiota analysis can be affected by the choice of PCR primers, sequencing platforms, and analytical software (Fouhy et al., 2016; Marizzoni et al., 2020). To validate the effects of analytical software (QIIME2 vs. LAST) and sequencing region (i.e., V1–V9 near full-length vs. V3–V4 partial region), we extracted the V3–V4 region *in silico* from near full-length reads of MiniON and PacBio and analyzed them using the QIIME2 pipeline (Supplementary Figure 1A). The mock data set (*n* = 3) containing 10,000 reads of near full-length (V1–V9) were randomly picked from MinION and PacBio data in experiment 1. Then, V3–V4 region of 16S rRNA genes were extracted *in silico* from each V1–V9 mock data set to generate three sets of V3–V4 mock data (Mock 1–3; Supplementary Figure 1A). Mock data sets were analyzed using LAST and QIIME2 for taxonomic identification. In this validation, V3–V4 mock data set mimics sequence reads obtained by MiSeq platform.

## Experiment 2: Evaluation of MinION Platform to Characterize Rumen Microbiota at Species-Level

### Sample Collection, Amplicon Sequencing, and Data Analysis

In experiment 2, 28 Japanese Black cattle (17.1 ± 4.1 months old, fattening period) reared in the experimental farm of the National Livestock Breeding Center, Ohu Station (Aomori, Japan), were used. Roughage (1.5–4.0 kg/day; rice straw, orchard grass hay, or wheat straw) and concentrated diet (7.0–10.5 kg/day) were offered twice daily at 08.30 and 16.00 h. Rumen contents were collected from individual cattle using a stomach tube, 4 h after the morning feeding. The collected rumen contents were stored at −80°C until use without separating the solid and liquid fractions. Microbial DNA extraction, purification, amplicon sequencing using MiSeq and MinION, and downstream analyses were performed as described in experiment 1; data obtained from the MiSeq and MinION platforms were analyzed using the QIIME2 and the LAST aligner, respectively.

## Experiment 3: Validation of Applicability of Combination of MinION Platform and Buccal Swab Samples to Characterize Rumen Microbiota

### Sample Collection

In experiment 3, 30 Japanese Black cattle (15.0 ± 0.6 months old, fattening period) reared in the experimental farm of the National Livestock Breeding Center, Ohu Station (Aomori, Japan), were used. Timothy hay (3.0 kg/day) and concentrated diet (8.0 kg/day) were fed once daily at 10.00 h. The animals had free access to mineral blocks and water. Buccal swab samples were collected from individual animals as mentioned in experiment 1. For the reference sample of rumen microbiota, rumen contents were collected immediately after buccal swab sampling from five of the same animals using a stomach tube.

### Modification of PCR Conditions for MinION Library Preparation

Microbial DNA was obtained and used for PCR amplification of the near full-length 16S rRNA gene using primers 27F and 1525R as mentioned in experiment 1. However, most of samples were not amplified, possibly due to low concentration of DNA. The PCR conditions were thus modified as follows: initial denaturation at 94°C for 1 min, 20 cycles (originally 5 cycles) at 94°C for 30 s, 63°C for 30 s, and 65°C for 90 s, 15 cycles (originally 30 cycles) at 94°C for 30 s, 62°C for 30 s, and 65°C for 90 s, and a final extension step at 65°C for 5 min. We confirmed that the modified PCR conditions did not influence bacterial composition of eight rumen samples obtained in experiment 2; bacterial compositions in the identical sample were well correlated between original and modified PCR conditions (Supplementary Figure 2). Even using modified PCR conditions, 12 of 30 samples were failed in PCR amplification for MinION library preparation. To clarify the possible cause, we performed real-time PCR quantification of 16S rRNA gene by using bacterial universal primers (Supplementary Figure 3). The samples that failure amplification in PCR for MinION library preparation had a lower copy number of 16S rRNA genes. Based on this result, we attributed poor PCR amplification to a low DNA concentration in buccal swab samples used in experiment 3 rather than PCR inhibition.

### Amplicon Sequencing and Data Analysis

Eighteen buccal swabs and five rumen contents showing sufficient PCR amplification were used for amplicon sequencing with MinION platform. Three buccal swab samples with less than 5,000 reads remaining after taxonomic classification were excluded from the data set, and 20 samples (15 buccal swab samples and five rumen contents) were finally used in the analyses. Downstream analyses were performed using the LAST aligner as mentioned in experiment 1.

## Statistical Analysis

All statistical analyses were performed using R (v3.6.2). In experiment 1, the relative abundance of genus-level bacterial taxa was compared among MiSeq, MinION, and PacBio platforms using Dunnett’s test for multiple comparisons. Pearson correlation was used to compare the microbial composition between rumen contents and buccal swab samples at the respective sequencing platforms. In experiments 2 and 3, the Euclidean distance matrix was calculated based on the relative abundances of bacterial taxa in each sample and clustered using Ward’s method. The Pearson correlation among the relative abundance of the bacterial taxa was analyzed using the corrplot package version 0.84 (Wei and Viliam, 2017) in R. The differences in the relative abundances of the selected bacterial groups were evaluated using Welch’s *t*-test. Statistical significance was set at *p* < 0.05. In experiment 3, principal coordinate analysis was used to compare the microbial community structure based on the Bray–Curtis dissimilarity matrices using the vegan package v.2.5.6 (Oksanen et al., 2015) in R.

## Results

### Comparison of Resolution of Taxonomic Assignment and Microbial Composition Among Different Sequencing Platforms (Experiment 1)

**Figure 1:**
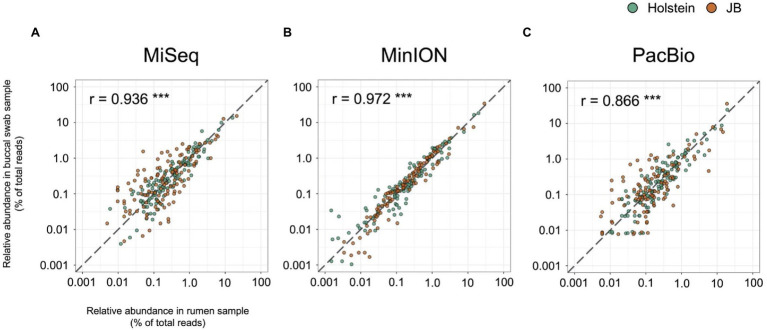
Correlation of relative abundance of genus-level taxa in the rumen samples and buccal swab samples determined using **(A)** MiSeq, **(B)** MinION, and **(C)** PacBio platforms. Individual plots represent respective bacterial taxa with relative abundance of >0.1% in at least one platform in either the rumen or buccal swab of either cattle breed (Holstein or Japanese Black). Different colors indicate the cattle breed (Holstein, green; Japanese Black, orange). The dashed lines indicate slope of 1, which indicate numerical coincidence of relative abundances in the rumen and buccal swab samples. Pearson correlation value (r) was calculated for the respective platforms. ^***^p < 0.001.

**Figure 2:**
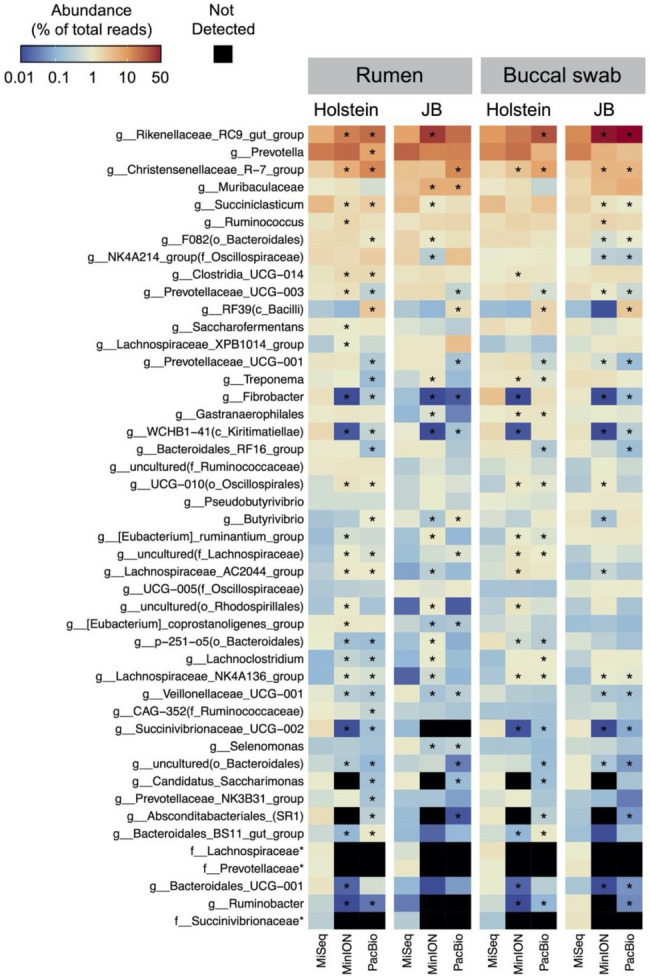
Distribution of the major genus-level taxa determined using MiSeq, MinION, and PacBio platforms. Only the taxa with relative abundance of >1% in at least one platform in either the rumen or buccal swab of either cattle breeds (Holstein or Japanese Black) were included in the heat map. The scale colors indicate the relative abundances. Cells with asterisks in MinION and PacBio indicate significant (*p* < 0.05, Dunnett’s test) differences in means compared to MiSeq. Name of taxa prefixed “f_” indicates unclassified bacterial family.

Table 1 shows the results of taxonomic assignments determined using MiSeq, MinION, and PacBio platforms. All reads derived from the MinION and PacBio platforms were classified at the species-level. In contrast, 94 and 58% of the reads derived from the MiSeq platform were classified at the genus- and species-level, respectively. Based on these results, all three platforms were considered capable of performing taxonomic classification at the genus-level. Therefore, to compare the results in parallel among different platforms, the microbial composition in experiment 1 was analyzed at the genus-level. In total, 154 genera with relative abundance of >0.1% in at least one platform in either the rumen or buccal swab were considered to be observed and were used for the analysis (Supplementary Data 1). A comparison of the relative abundance of the genus-level taxa in the rumen and buccal swab samples is shown in Figure 1. A significant (*p* < 0.001) correlation in the relative abundance of bacterial taxa was confirmed between rumen contents and buccal swab samples, irrespective of the sequencing platform used (Figure 1). Figure 2 shows the distribution of major genus-level taxa determined using the three sequencing platforms. Although Rikenellaceae RC9 gut group, *Prevotella* spp., and Christensenellaceae R-7 groups were predominant, the relative abundance of various taxa differed significantly (*p* < 0.05) among the sequencing platforms. We speculated that sequencing region and/or analytical software influence the taxonomic identification. These possibilities were validated using the mock data set (Supplementary Figure 1). Taxonomic identification at family-level was similar regardless of analyzed region and analytical software (Supplementary Figures 1B–E). In contrast, QIIME tended to fail taxonomic identification at genus- and species-level compared to LAST (Supplementary Figures 1B,C).

**Table 1:** Summary of amplicon sequencing results derived from three sequencing platforms in experiment 1.

Item	Sequencing platform		
	MiSeq + QIIME2	MinION + LAST	PacBio + LAST
Library preparation			
Target region of 16S rRNA gene for amplification	V3–V4	V1–V9	V1–V9
Primer sets	341F, 805R	27F, 1525R	27F, 1492R
Number of reads used for analysis			
Total input reads	342,469	343,013	65,158
Total taxa-assigned reads^1^	227,748	277,407	45,809
Average remained reads per sample	18,979	23,117	3,817
Reads assigned to taxa (%)^2^			
Order	225,384 (99.0)	277,407 (100)	45,809 (100)
Family	224,566 (98.6)	277,407 (100)	45,809 (100)
Genus	214,527 (94.2)	277,407 (100)	45,809 (100)
Species	132,393 (58.1)	277,407 (100)	45,809 (100)

1 Reads that were successfully assigned to any taxa in Silva 138 99% reference sequences.

2 Proportion of the number of reads successfully assigned to taxa at respective taxonomic levels against total number of taxa-assigned reads.

### Evaluation of MinION Platform to Characterize Rumen Microbiota at Species-Level (Experiment 2)

**Figure 3:**
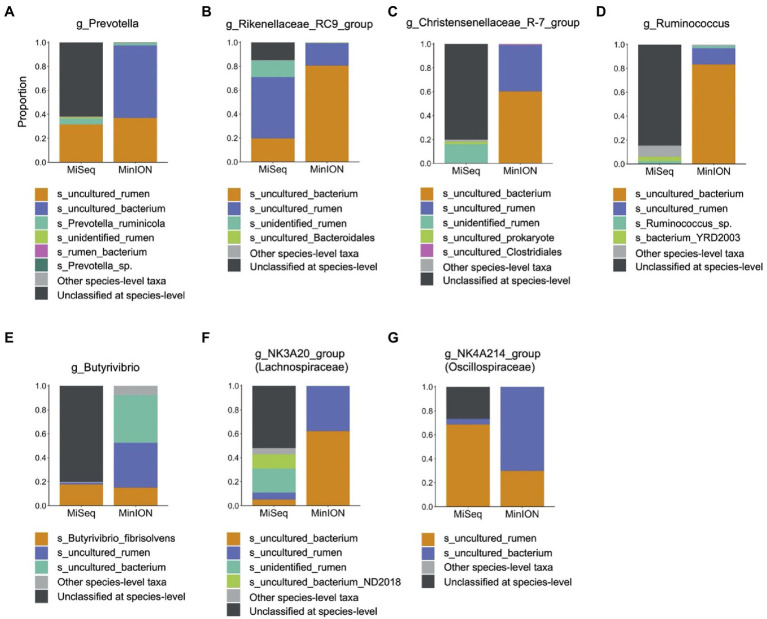
Composition of predominant bacterial taxa at species-level analyzed using MiSeq and MinION platforms. Proportions of each species-level taxa were calculated for **(A)** *Prevotella* spp., **(B)** Rikenellaceae RC9 gut group, **(C)** Christensenellaceae R-7 group, **(D)** *Ruminococcus* spp., **(E)** *Butyrivibrio* spp., **(F)** Lachnospiraceae NK3A20 group, and **(G)** Oscillospiraceae NK4A214 group. Only species-level taxa with relative abundances of >0.01% in at least 14 individuals (half of animals used in experiment) in either MiSeq or MinION were included as major species-level taxa, and other minor taxa were merged into “Other species-level taxa.” Black bar indicates unclassified at species-level.

In experiment 2, 32 family-level taxa and 66 genus-level taxa with relative abundance of >0.1% in at least 14 individuals (half of the herd) in either MiSeq or MinION platforms were considered to be observed and were used for the analysis (Supplementary Data 2). Table 2 shows the predominant bacterial families and genera identified using the MiSeq and MinION platforms. The six most abundant bacterial families, namely, Prevotellaceae, Rikenellaceae, Christensenellaceae, Ruminococcaceae, Lachnospiraceae, and Oscillospiraceae, were shared between the sequencing platforms, although the relative abundances of several bacterial taxa, particularly those of Rikenellaceae, differed significantly (*p* < 0.001) between the sequencing platforms (Table 2). At the genus-level, *Prevotella* spp., Rikenellaceae RC9 gut group, Chrsitensenellaceae R-7 group, *Ruminococcus* spp., Lachnspiraceae NK3A20 group, *Butyrivibrio* spp., and Oscillospiraceae NK4A214 groups were found to be the predominant bacterial groups on both sequencing platforms. Figure 3 shows the species-level composition of the predominant genus-level taxa identified using MiSeq and MinION platforms. All reads derived from the MinION platform were successfully classified at the species-level, whereas 20–90% of the reads derived from MiSeq were not classified at the species-level. Assignment of the species-level taxa in the MinION platform showed that the predominant genus-level taxa were mainly composed of uncultured bacterial taxa (i.e., s_uncultured_bacteria and s_uncultured_rumen).

**Table 2:** Relative abundance of predominant rumen bacterial taxa in Japanese Black cattle at family- and genus-level identified using MiSeq and MinION platforms in experiment 2.

Taxa^1^	MiSeq	MinION	Pooled SEM	*p*-value^2^
f__Prevotellaceae	29.217	28.877	2.087	0.936
g__Prevotella	24.908	25.687	2.006	0.851
g__Prevotellaceae_UCG-001	1.021	1.699	0.209	0.113
g__Prevotellaceae_UCG-003	1.038	0.709	0.073	0.026
g__Prevotellaceae_NK3B31_group	0.334	0.446	0.050	0.278
Unclassified at genus-level	1.540	ND	0.143	NA
f__Rikenellaceae	6.681	28.317	1.894	<0.001
g__Rikenellaceae_RC9_gut_group	5.914	26.209	1.775	<0.001
g__U29-B03	0.698	2.105	0.216	0.001
f__Christensenellaceae	6.806	8.313	0.580	0.198
g__Christensenellaceae_R-7_group	6.740	8.203	0.563	0.206
g__uncultured (Christensenellaceae)	0.054	0.110	0.010	0.008
f__Ruminococcaceae	7.171	7.898	0.606	0.554
g__Ruminococcus	2.818	4.842	0.408	0.014
g__UCG-001 (Ruminococcaceae)	2.307	2.299	0.313	0.991
g__uncultured (Ruminococcaceae)	0.325	0.367	0.065	0.756
g__CAG-352 (Ruminococcaceae)	0.220	0.253	0.029	0.581
Unclassified at genus-level	1.414	ND	0.141	NA
f__Lachnospiraceae	7.716	7.688	0.576	0.981
g__uncultured (Lachnospiraceae)	0.571	1.391	0.402	0.322
g__Lachnospiraceae_NK3A20_group	1.019	1.348	0.134	0.232
g__Lachnospiraceae_XPB1014_group	0.821	1.016	0.160	0.556
g__Marvinbryantia	0.334	0.583	0.065	0.063
g__Lachnospiraceae_AC2044_group	0.070	0.382	0.032	<0.001
g__[Ruminococcus]_gauvreauii_group	0.173	0.370	0.034	0.004
g__[Eubacterium]_ruminantium_group	0.241	0.254	0.026	0.795
g__[Eubacterium]_ventriosum_group	0.224	0.217	0.052	0.954
g__Butyrivibrio	1.383	0.211	0.124	<0.001
g__Lachnospiraceae_UCG-008	0.236	0.202	0.028	0.547
g__Syntrophococcus	0.047	0.159	0.014	<0.001
g__Pseudobutyrivibrio	0.278	0.142	0.045	0.143
g__Roseburia	0.025	0.113	0.013	<0.001
g__Acetitomaculum	0.252	0.093	0.023	<0.001
Unclassified at genus-level	1.323	ND	0.109	NA
f__Oscillospiraceae	8.523	4.005	0.544	<0.001
g__NK4A214_group	6.324	2.490	0.386	<0.001
g__Colidextribacter	0.888	0.594	0.112	0.202
g__UCG-005 (Oscillospiraceae)	0.540	0.426	0.068	0.416
g__UCG-002 (Oscillospiraceae)	0.309	0.228	0.044	0.372
g__V9D2013_group	0.235	0.149	0.032	0.193
g__uncultured (Oscillospiraceae)	0.174	0.085	0.016	0.007

1 The abundance of major bacterial families showing relative abundance of >5% in at least 14 individuals (half of the animals used in the experiment) in either the MiSeq or MinION platforms and genus-level taxa belonging to major bacterial families with relative abundance of >0.1% in at least 14 individuals in either the MiSeq or MinION platforms were listed.

2 *p*-values were calculated based on Welch’s *t*-test.

### Investigating Individual Differences in Rumen Bacterial Compositions at Species-Level Using MinION Platform (Experiment 2)

**Figure 4:**
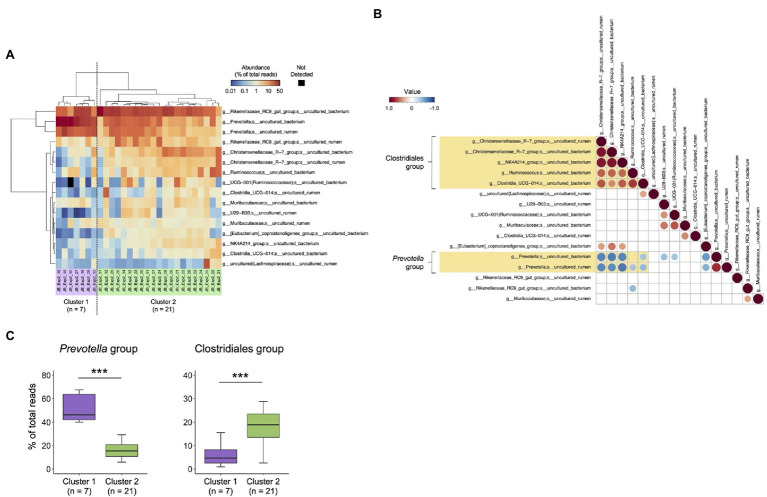
Characterization of rumen bacterial community structure in Japanese Black cattle using MinION platform. **(A)** Heat map with hierarchical clustering based on relative abundances of major species-level taxa. The relative abundances of species-level taxa showing >1% average relative abundance were included in the heat map and were used for hierarchical clustering based on the combination of Euclidean distance matrix and Ward’s clustering method. The scale colors indicate the relative abundances. Animals were grouped into two clusters (cluster 1, *n* = 7 and cluster 2, *n* = 21). **(B)** Pearson correlations among the abundance of major species-level taxa. The species-level taxa shown in **(A)** were included in the matrix. The values of relative abundance (% of the total reads) of respective bacterial taxa were used for calculations. Matrices were clustered using the “hclust” method and visualized in “corrplot” R packages. Only the plots showing statistical significance (*p* < 0.05) are indicated in the panel. The scale colors indicate the correlation: positive (closer to 1: red circle) or negative (closer to −1: blue circle). **(C)** Comparison of the relative abundance of Clostridiales and *Prevotella* groups in cluster 1 and cluster 2, which are determined using cluster analysis shown in **(B)**. ^***^p < 0.001 (Welch’s *t*-test).

To investigate the individual differences in rumen bacterial composition, 16 major species-level taxa with mean relative abundance > 1% determined using the MinION platform were used for the cluster analysis (Figure 4A). Based on the distribution patterns of the major species-level taxa, 28 animals were divided into two clusters (cluster 1, *n* = 7; cluster 2, *n* = 21). Correlation analysis of the relative abundance of major species-level taxa (Figure 4B) showed that the abundance of two uncultured *Prevotella* (hereinafter, referred to as *Prevotella* group) showed negative correlation (*p* < 0.05) with those of five Clostridiales bacteria (hereinafter, referred to as Clostridiales group), including two uncultured Christensenellaceae R-7 group, uncultured NK4A214 group, uncultured *Ruminococcus* spp., and uncultured Clostridia UCG-014 group. The distribution of species-level taxa within Clostridiales and *Prevotella* groups correlated positively (*p* < 0.05) with each other. Figure 4C shows the comparison of the relative abundance of Clostridiales and *Prevotella* groups in cluster 1 and cluster 2, as determined using the cluster analysis shown in Figure 4A. Cluster 1 had higher proportion of *Prevotella* group than cluster 2, whereas cluster 2 had higher proportion of Clostridiales group than cluster 1.

### Validation of Applicability of Combination of MinION Platform and Buccal Swab Samples to Characterize Rumen Microbiota (Experiment 3)

**Figure 5:**
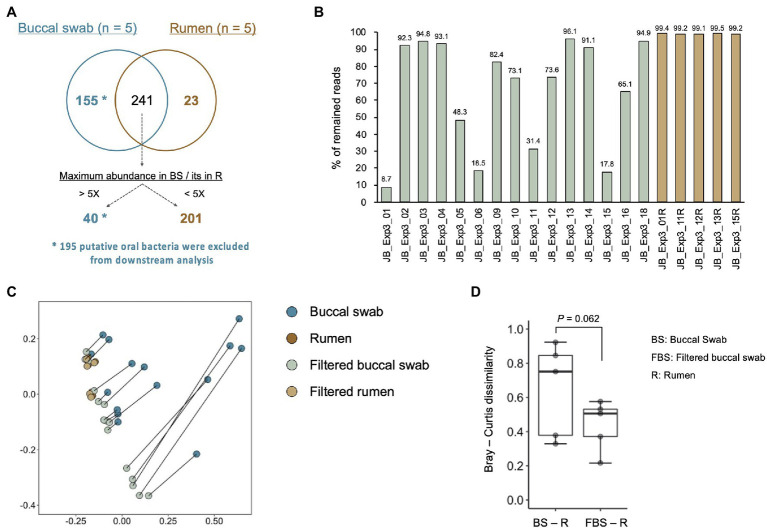
Evaluation of oral bacterial contamination and its removal from buccal swab samples. **(A)** Comparison of identified bacterial taxa between buccal swab and rumen sample collected from the same animals. Bacterial taxa, which were detected only in the buccal swab sample (*n* = 155) and the taxa showing five times greater abundance in buccal swab sample than in rumen sample (*n* = 40), were determined as putative oral bacteria. After removal of putative oral bacteria (*n* = 195), 224 species-level taxa (23 taxa found only in rumen samples and 201 taxa detected from both samples) remained. **(B)** The proportion of reads remaining after removal of the putative oral bacteria. **(C)** Effect of filtering out the putative oral bacteria on the bacterial composition in the buccal swab and rumen samples. Principal coordinate analysis on Bray–Curtis dissimilarities was used for comparing bacterial composition. Different colors indicate the data set (original buccal swab, blue; original rumen, brown; filtered buccal swab, light green; and filtered rumen, light orange). Plots of original buccal swab and filtered buccal swab samples collected from the same animals were connected by gray lines. **(D)** Comparison of Bray–Curtis dissimilarities for buccal swab samples/rumen contents and filtered buccal swab samples/rumen contents.

In experiment 3, we modified PCR conditions from that for experiments 1 and 2 due to poor amplification of 16S rRNA genes derived from buccal swab samples (Supplementary Figures 2, 3). We confirmed that PCR conditions for MinION library preparation did not influence the bacterial composition, and modified PCR conditions were used in experiment 3. In total, 419 species-level taxa with relative abundance of >0.1% in at least five of 20 samples (five rumen contents and 15 buccal swab samples) were considered to be observed and were used for the analysis (Supplementary Data 3). To determine the oral bacteria that to be filtered out from the buccal swab samples, the bacterial composition of the rumen contents and buccal swab samples from the same five animals were compared (Figure 5). In total, 155 bacterial taxa detected only in buccal swab samples were considered to be putative oral bacteria (Figure 5A). In addition, 40 of 241 shared bacterial taxa between the buccal swab and rumen samples were identified as putative oral bacteria according to the following criteria: bacterial taxa, maximum relative abundance of which in the buccal swab sample was more than five times higher than that in the rumen, were considered putative oral bacteria. Finally, 195 putative oral bacterial taxa were excluded from the data sets, and 224 species-level taxa (23 taxa found only in the rumen sample and 201 taxa detected in both samples) remained. The proportion of reads remaining after the exclusion of putative oral bacteria is shown in Figure 5B. The proportion of excluded putative oral bacteria ranged from 5 to 90% (mean, 34.5%; median, 26.4%) of the total reads in buccal swab samples, whereas it was less than 1% in rumen samples. Principal coordinate analysis was performed to evaluate the effect of filtering out of oral bacteria on the similarity of bacterial composition between rumen and buccal swab samples (Figure 5C). The filtered buccal swab sample was closer to the rumen samples than the original buccal swab sample (Figure 5D). However, the five samples that were highly contaminated with oral bacteria (sample ID JB_Exp3_01, 05, 06, 11, and 15 in Figure 5B) were still separated from the rumen samples even after removal of the putative oral bacteria (Figure 5C). Thus, these five samples were excluded from downstream analysis.

### Evaluation of Individuality of Rumen Microbiota at Species-Level Taxa Using Combination of Buccal Swab Sample and MinION Platform (Experiment 3)

**Figure 6:**
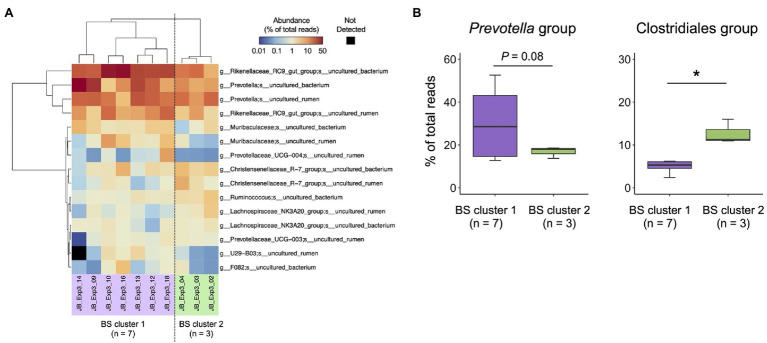
Characterization of rumen bacterial community structure in Japanese Black cattle using a combination of buccal swab sample and MinION platform **(A)** Heat map with hierarchical clustering based on relative abundance of major species-level taxa in the rumen of Japanese Black cattle. The relative abundance of species-level taxa showing >1% average relative abundance were subjected to hierarchical clustering based on the combination of Euclidean distance matrix and Ward’s clustering method. Animals were grouped into two clusters (BS cluster 1, *n* = 7 and BS cluster 2, *n* = 3). **(B)** Comparison of relative abundance in Clostridiales group and *Prevotella* group suggested in experiment 2 (Figure 4B). Box colors indicate the animal clusters suggested in **(A)** BS cluster 1 (purple), BS cluster 2 (green). ^*^p < 0.05 (Welch’s *t*-test).

Individual differences in the bacterial composition of the 10 filtered buccal swab samples were evaluated using cluster analysis (Figure 6A). Fifteen major species-level taxa with average relative abundance of >1% were used for the cluster analysis, and 10 animals were divided into two clusters buccal swab [(BS) cluster 1, *n* = 7; BS cluster 2, *n* = 3]. Based on the individual differences in rumen microbiota observed in experiment 2 (Figure 4), the abundances of the *Prevotella* and Clostridiales groups were compared between BS cluster 1 and BS cluster 2 (Figure 6B). BS cluster 1 had higher proportion of *Prevotella* group, whereas BS cluster 2 had higher proportion of Clostridiales group.

## Discussion

To determine the key rumen microbes influencing host productivity, a method for high-throughput rumen microbial profiling, which can be used for large-scale studies, is required. In addition, to clarify the function of the key players, the resolution of taxonomic assignment needs to be increased from the genus to species-level. The present study aimed to establish a method for high-resolution and high-throughput rumen microbiota analysis using a combination of MinION sequencing platform and buccal swab samples, which act as a proxy for rumen contents.

In experiment 1, the resolution of taxonomic assignment and microbial composition were compared among three different analytical workflows: the V3–V4 partial regions of the 16S rRNA gene were sequenced using the widely used MiSeq platform, and the near full-length (V1–V9 region) 16S rRNA genes were sequenced using MinION or PacBio platforms. Although the sequencing accuracy of MinION and PacBio platforms is lower than MiSeq, sequencing of multiple variable regions of 16S rRNA genes complements the lower accuracy of these platforms. However, the OTU-based bioinformatics pipeline used for MiSeq platforms, such as QIIME, is not suitable for analyzing the sequencing data with a high error rate (Ma et al., 2017; Santos et al., 2020). In the present study, we performed taxonomic identification of MinION and PacBio data using the LAST aligner, which enables alignment of sequencing reads to the references by calculating an alignment probability for every possible pair of aligned bases (Frith et al., 2010). This tool has been previously applied to microbiota studies using MinION (Quick et al., 2014; Shin et al., 2016; Xia et al., 2017; Nygaard et al., 2020). Studies have shown that longer sequenced regions of 16S rRNA genes enable taxonomic assignment at higher resolution than short sequence reads derived from the MiSeq platform (Myer et al., 2016; Nygaard et al., 2020; Matsuo et al., 2021). In the present study, MinION and PacBio platforms succeeded in identifying the microbial composition at the species-level. In addition, the microbial composition of the rumen contents and buccal swab samples showed good correlation. Therefore, a combination of buccal swab samples and amplicon sequencing near the full-length 16S rRNA gene enables high-resolution and high-throughput profiling of rumen microbiota. Although both MinION and PacBio platforms can be applied for rumen microbiota analysis, the MinION platform appears to be better than the PacBio platform for large-scale analysis because of its higher throughput and lower cost.

Although full-length sequencing of 16S rRNA gene showed higher resolution of taxonomic identification compared to short-read sequencing, we observed significant differences in the relative abundance of the predominant bacterial taxa among the three platforms. Validation with mock data set in the present study indicates that sequencing region and analytical software are unlikely to influence the family-level identification, whereas genus- or species-level identification seems to be affected by the combination of the sequencing region and analytical software; QIIME2 tended to fail taxonomic identification regardless of read length. The combination of MiSeq sequencer and QIIME has been widely used as a standard tool for microbial ecology research owing to robust and accurate taxonomic identification using Naïve Bayes classifier and bootstrap validation in QIIME2. However, this approach is not free from the limitation; the previous study indicated that the Naïve Bayes classifier struggled for taxonomic identification at genus-level (McGovern et al., 2018). On the other hand, the LAST aligner for long read with a high error rate prioritizes the alignment sensitivity to assign the taxonomic information by lowering mismatch and gap costs (Shin et al., 2016; Nygaard et al., 2020). The alignment-based approach is less accurate for taxonomic identification than QIIME, and the average percent identity against the reference sequence determined by the LAST aligner in the present study was 92.88 ± 3.49% (mean ± SD, *n* = 955,780; Supplementary Figure 4). In addition, the LAST aligner picks the reference sequence showing the highest similarity for taxon assignment. Therefore, taxonomic assignment by the LAST aligner is equivalent to determining the closest relatives of the query sequence rather than identifying true taxon. Further comparative investigation among analytical software is needed to validate not only the resolution but also the accuracy of taxonomic identification. Taken together, our results indicate the limitation of amplicon sequencing for the determination of “true microbiota.” For the identification of key rumen bacteria that can improve host productivity, the results of amplicon sequencing have to be validated using different analyses, such as shotgun metagenome sequencing and/or quantitative PCR.

In experiment 2, the MinION platform was evaluated using this approach for the analysis of rumen samples. The rumen samples of Japanese Back cattle were used for the evaluation of the MinION platform because we have previously identified the core rumen bacterial members of this breed using the MiSeq platform: *Prevotella* spp., unclassified Clostridiales, unclassified Ruminococceae, unclassified Bacteroidales, and *Ruminococcus* spp. occupied approximately 70% of the rumen microbiota (Miura et al., 2021). Although the relative abundances of several bacterial taxa, particularly those of Rikenellaceae, differed between the sequencing platforms as discussed above, the predominant bacterial families and genera determined using the MinION platform were consistent with the previous results obtained using the MiSeq platform. In addition, the MinION platform succeeded in further identifying the species-level composition of core rumen bacterial members, which consist mainly of uncultured bacterial groups. Kim et al. (2011) reported the predominance of uncultured bacteria in the rumen, and therefore, attempts have been made to cultivate uncultured rumen bacteria (Koike et al., 2010; Kenters et al., 2011; Nyonyo et al., 2013). Nevertheless, the number of cultivated bacterial strains is still insufficient, and the necessity for the cultivation of novel isolates has been emphasized to understand the function of the core rumen microbiome and to expand genome references (Creevey et al., 2014). The identification of rumen bacterial composition at the species-level using MinION is useful for clarifying the target bacterial group for cultivation to reveal the functions of key players in rumen fermentation.

In a previous study, we have demonstrated that the abundance of *Prevotella* spp. correlated negatively with that of Ruminococcaceae and Christensenellaceae in the rumen of Japanese Black cattle (Miura et al., 2021). This relationship was confirmed at the species-level taxa in the present study, although all species-level taxa consisted of uncultured bacteria. Furthermore, high-resolution analysis using the MinION platform revealed individual differences in the rumen bacterial composition of Japanese Black cattle, and the rumen microbiota was divided into two types: one showed higher abundance of uncultured *Prevotella* spp. than of uncultured Christensenellaceae R-7 group/other Clostridiales taxa, whereas the other showed the opposite distribution of these bacterial taxa (i.e., lower *Prevotella* spp. and higher Christensenellaceae R-7/other Clostridiales). A study suggested that the abundance of *Prevotella* spp. correlated positively with milk yield in dairy cows (Xue et al., 2020). The Christensenellaceae R-7 group has been recently recognized as an important bacterial group that produces hydrogen, which is utilized for methane production in the rumen (Greening et al., 2019). Therefore, differences in the abundance of these bacterial taxa in the rumen may influence host productivity.

Recent studies have validated the buccal swab samples as a proxy of rumen contents for the purpose of applying them to large-scale analysis of rumen microbiota (Kittelmann et al., 2015; Tapio et al., 2016; Young et al., 2020; Amin et al., 2021). In the present study, although the applicability of a combination of MinION platform and buccal swab samples was demonstrated in experiment 1, it was validated again in experiment 3 using more animals. Previous studies have reported the presence of oral bacteria in buccal swab samples that have to be filtered out before analysis (Kittelmann et al., 2015; Young et al., 2020). In the present study, 195 species-level bacterial taxa showing an exclusively higher abundance in buccal swab samples were identified as putative oral bacteria. The ratio of putative oral bacteria to total bacteria in buccal swab samples ranged from 5 to 90% (mean, 34.5%; median, 26.4%), which was consistent with the results of previous studies that reported mean of 43% (Kittelmann et al., 2015) or 57% (Amin et al., 2021). Although filtering out of the putative oral bacterial taxa effectively improved the similarity in bacterial composition between buccal swab and rumen samples, the bacterial composition of five of 15 buccal swab samples, which were highly contaminated with oral bacteria, still differed from that of the rumen contents. Young et al. (2020) accurately identified oral bacterial taxa in buccal swab samples of Holstein dairy cows using a machine learning classifier. In the present study, we aimed to determine the distribution of core rumen microbes using buccal swabs and, therefore, used less-strict criterion to remove major oral bacteria. As shown by Kittelmann et al. (2015), the differences between oral microbiota and rumen microbiota should be investigated in detail using ruminants under different dietary conditions to further optimize the filtering method for sequencing data obtained from buccal swabs.

In experiment 3, we investigated the individual differences in bacterial composition at the species-level using filtered buccal swab samples. The predominant bacterial taxa, such as Rikenellaceae RC 9 gut group, uncultured *Prevotella* spp., and Christensenellaceae R-7 group, were also detected in the rumen contents in experiment 2. Although the number of samples used in the analysis was not sufficient, 10 animals were divided into two clusters based on the distribution patterns of the predominant bacterial taxa. Furthermore, these two clusters differed in the abundances of uncultured *Prevotella* and Clostridiales bacteria, as observed in experiment 2, in which rumen contents were used for the analysis. Based on these findings, we concluded that a combination of the MinION platform and buccal swab samples may be potentially used for the large-scale analysis of rumen microbiota.

The limitation of the present study was the number of animals collecting the buccal swabs and rumen contents simultaneously; six animals in experiment 1 and five animals in experiment 3. Ideally, rumen contents and buccal swabs needed to be sampled for all animals in experiments 2 and 3. However, it was unable due to the limitation on access to the production animals. We observed similar trends in the separate animal groups (experiments 2 and 3), suggesting the reproducibility of microbiota determination both in rumen contents and buccal swabs. Another limitation was PCR amplification in buccal swab samples; 12 of 30 buccal swab samples were not amplified in experiment 3, even after using the modified PCR conditions, possibly due to a low DNA concentration. Further optimization of DNA extraction and PCR conditions is required to improve the amplicon yield. We also acknowledge the limitation to the MinION platform combined with the LAST aligner for microbiota analysis. Because of the high error rate in the MinION sequencing and less power for taxon assignment in the LAST aligner, the improved resolution of microbiota analysis by the MinION platform does not necessarily ensure the accurate determination of bacterial taxa in microbiota. To achieve species-level identification in robust and accurate manner, sequencing technology and analytical pipeline, including the reference database, need to be updated.

This study evaluated a combination of the MinION platform and buccal swab samples for improving the resolution and throughput of rumen microbiota analysis. We confirmed that the MinION amplicon sequencing of near full-length 16S rRNA genes enabled species-level taxonomic identification of rumen microbiota using buccal swab samples. Although further technical updates and validations are required for robust and accurate taxonomic identification, a combination of the MinION platform and buccal swab samples may be potentially applied for rumen microbial analysis in large-scale studies.

## Data Availability Statement

The datasets presented in this study can be found in online repositories. The names of the repository/repositories and accession number(s) can be found at: https://www.ncbi.nlm.nih.gov/genbank/, PRJNA765156.

## Author Contributions

SK designed the study. HM, MT, YO, GE, YM, KIt, YN, KIw, TM, and SK performed the sampling. HM and MY performed all lab work and data acquisition. HM and SK analyzed the data. HM, YS, YK, and SK interpreted the data. HM and SK drafted the manuscript. All authors contributed to the article and approved the submitted version.

## Funding

This work was supported by JSPS KAKENHI grant numbers 17K19315 to SK and 21J13430 to HM. This work was also supported partially by the Cabinet Office, Government of Japan, Cross-ministerial Moonshot Agriculture, Forestry and Fisheries Research and Development Program, “Technologies for Smart Bio-industry and Agriculture” (no. JPJ009237).

## Conflict of Interest

YO and KIw are affiliated with MEIJI FEED. CO., Ltd.

The remaining authors declare that the research was conducted in the absence of any commercial or financial relationships that could be construed as a potential conflict of interest.

## Publisher’s Note

All claims expressed in this article are solely those of the authors and do not necessarily represent those of their affiliated organizations, or those of the publisher, the editors and the reviewers. Any product that may be evaluated in this article, or claim that may be made by its manufacturer, is not guaranteed or endorsed by the publisher.
