# Supplementary material for: Application of MinION Amplicon Sequencing to Buccal Swab Samples for Improving Resolution and Throughput of Rumen Microbiota Analysis
Source: Front Microbiol. 2022 Mar 24;13:783058. doi: 10.3389/fmicb.2022.783058 (PMC8989143; doi:10.3389/fmicb.2022.783058)
Supplement: Supplementary file 1 [file Data_Sheet_1.docx]

Supplementary Material

**
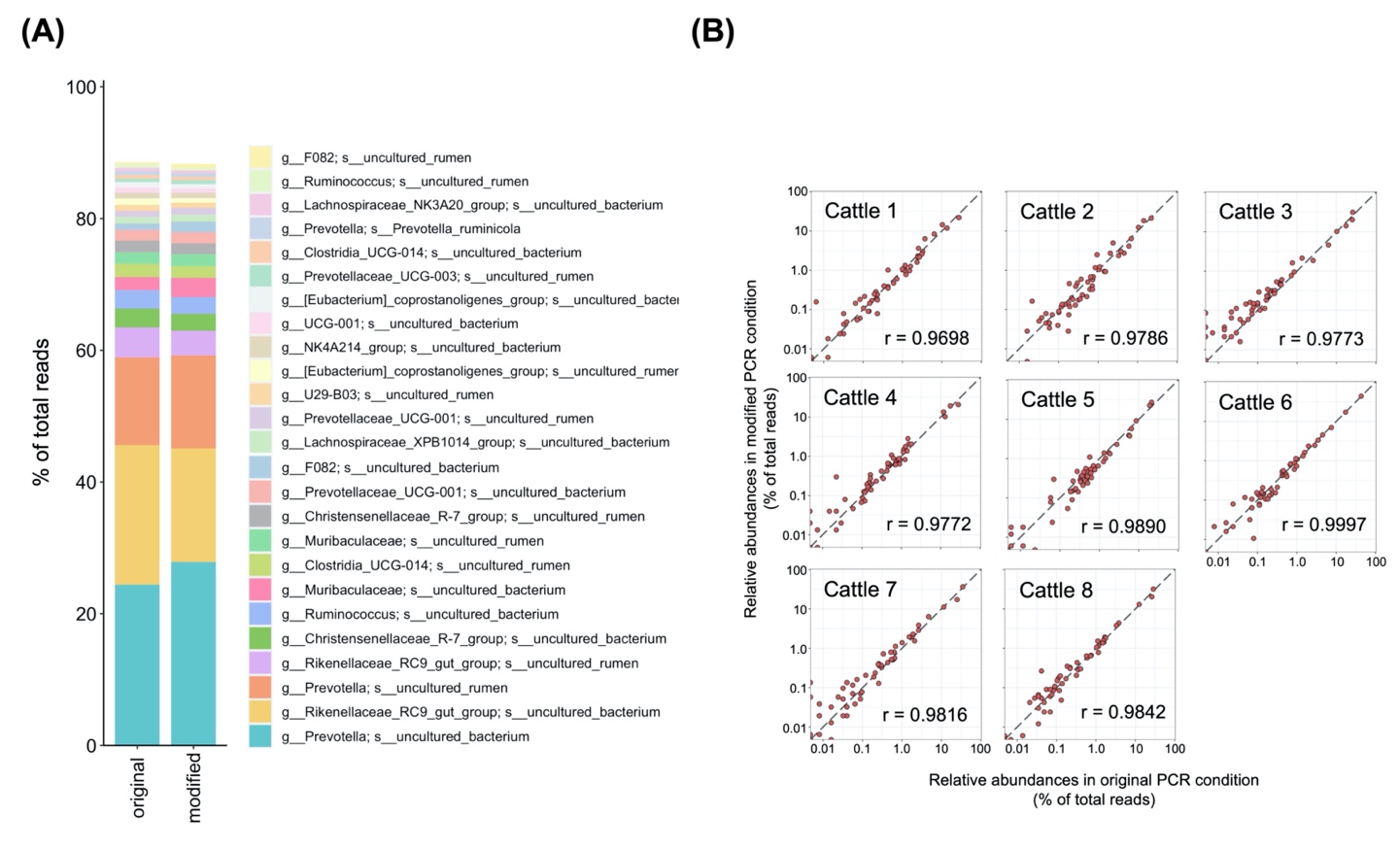
**

**Supplementary Figure 2. Validation of modified PCR conditions for MinION library preparation.** The same rumen samples (n = 8) were used for PCR amplification with original conditions used in experiment 2 and modified conditions. (**A**) Major bacterial taxa at species level showing > 0.5% average relative abundance. (**B**) Correlation of identified bacterial taxa at species level between original and modified PCR conditions for MinION library preparation. Individual plots represent respective bacterial taxa shown in the bar chart. Pearson correlation value (r) was calculated for each sample.


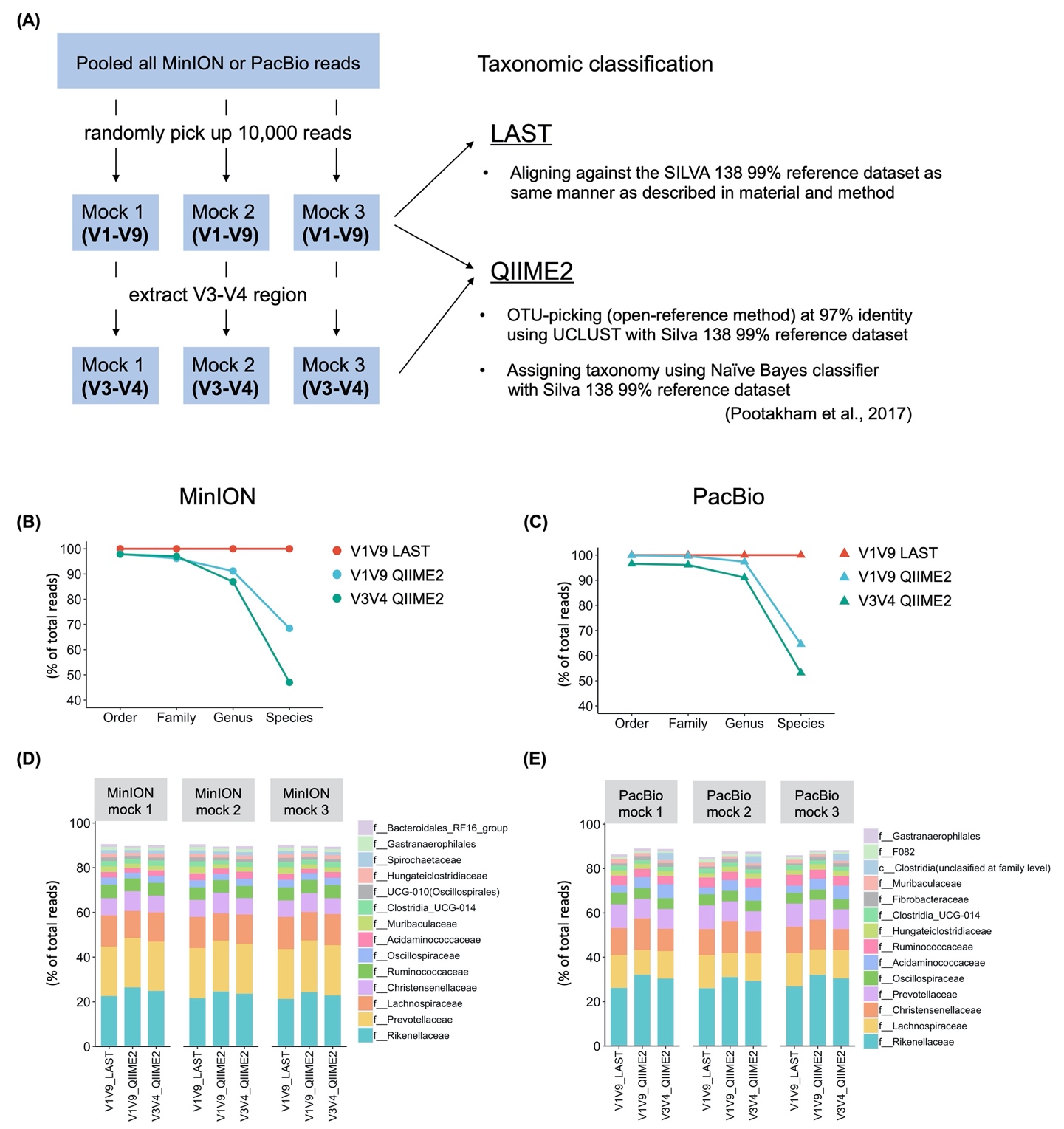


**Supplementary Figure 1. Validation of analyzed region of 16S rRNA gene and analytical software.** (**A**) Workflow of the validation study. The mock data set (n = 3) containing 10,000 reads of near full-length (V1−V9) were randomly picked from MinION and PacBio data in experiment 1. Then, V3−V4 region of 16S rRNA genes were extracted *in silico* from V1−V9 mock data to generate V3−V4 mock data. Both mock data were analyzed using LAST and QIIME2. Line graphs show the resolution of taxonomic classification of mock data from (**B**) MinION and (**C**) PacBio. The values indicate the proportion of reads successfully assigned to the taxa at each taxonomic level. Different colors indicate analyzed region of 16S rRNA gene and analytical software used (V1−V9 analyzed using LAST, red; V1−V9 analyzed using QIIME2, light blue; and V3−V4 analyzed using QIIME2, green). Stacked bar graphs show the relative abundance of major family level taxa with an average relative abundance of > 1% in (**D**) MinION and (**E**) PacBio.


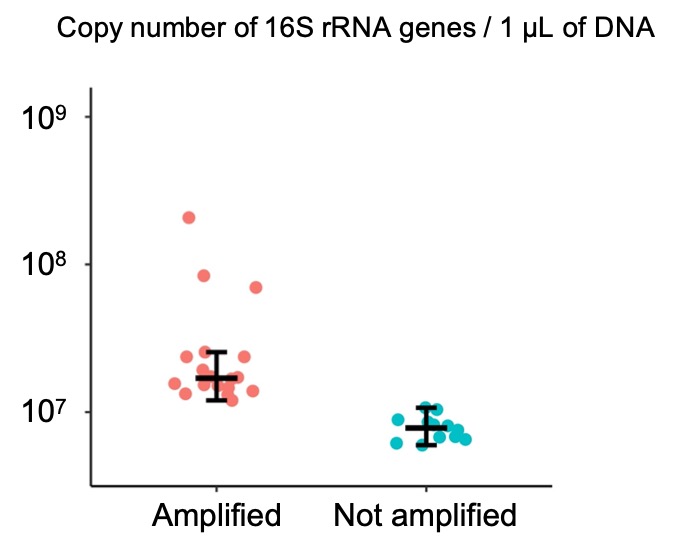


**Supplementary Figure 3. Copy number of total bacterial 16S rRNA genes per microliter of template DNA in buccal swab sample.** The red and blue dots indicate amplified samples (n = 18) and non-amplified samples (n = 12) in MinION library preparation in experiment 3, respectively. Quantitative real-time PCR was performed using a LightCycler system with a KAPA SYBR Fast qPCR kit (Kapa Biosystems, Charlestown, USA) with the primer set of 341F (5′-CCTACGGGAGGCAGCAG-3′) and 534R (5′- ATTACCGCGGCTGCTGG -3′) (Muyzer et al. 1993). The PCR thermal conditions and PCR mixture were identical to those reported earlier (Myint et al., 2017).

**Supplementary Figure 4. Distribution of percent identities against reference sequences determined by the LAST aligner in the present study.** Violin plots show mean (white point), quartiles (black bars), and kernel density estimation (violin) for percent identity of reads assigned taxonomic information in the present study (n = 955,780).
